# Supplementary material for: Patient-reported physical functioning and quality of life after pelvic ring injury: A systematic review of the literature
Source: PLoS One. 2020 Jul 17;15(7):e0233226. doi: 10.1371/journal.pone.0233226 (PMC7367481; doi:10.1371/journal.pone.0233226)
Supplement: S1 File — (DOCX) [file pone.0233226.s001.docx]

**S1 Text.** Description of the included PROMs

*Majeed Pelvic Score (MPS)*The MPS is a physician-rated score to assess function after major pelvic ring injuries (66). The score consists of seven items divided into five subscales, pain, work, sitting, standing, (walking aids, gait unaided, walking distance), and sexual intercourse. The total amount of points that can be achieved is 100, or 80 in case patients were not working before the injury. Majeed suggested cut-off values for excellent, good, fair, and poor results in those working before the injury (respectively: >85, 70-84, 55-69, and<55 points) and those not working before the injury (respectively >70, 55-69, 45-54, and <45 points). Although the MPS demonstrated acceptable psychometric properties for outcome assessment in chronic sacroiliac joint pain (65), its validity for outcome assessment after pelvic ring injuries has not been established.

*Iowa Pelvic Score (IPS)*The IPS was developed by Templeman et al. (67). It consists of 25 items within six subscales: activities of daily living, work history, pain, limp, visual pain line, and cosmesis. The total amount of points that can be achieved is 100, with higher scores indicating better function. The validity of the IPS in patients with pelvic ring injuries has not been studied.
 *Pelvic Outcome Score (POS)*The so called ‘Becken outcome score’ or Pelvic Outcome Score was developed by Pohlemann et al. (68). It is a categorical scoring instrument, divided into three sections:1) radiological result,2) clinical result with rating of function, neurological, urological and sexual deficits, and 3) status of reintegration. The first and second sections are summarized as “pelvic outcome”. The validity for outcome assessment after pelvic ring injuries has not been established.

*Merle d’Aubigne-Postel Score*The Merle d’Aubigne-Postel Score was developed by d’Aubigne and Postel (69) for the evaluation of functional results after hip arthroplasty with acrylic prosthesis, and is therefore not a pelvic outcome score, although regularly used as such. The score is divided into three sections; pain, mobility, and ability to walk, each of which can score up to six points in case of the best possible situation. Clinical grades (very good, good, medium, fair, poor) are given by the scores of pain and walking ability and adjusted down one to two grades, depending on the mobility score. The score was found to be reliable (70) but not valid, for the use in patients with total hip arthroplasty. Also, its validity for the assessment of function of patients with pelvic ring injuries has not been established.

*Musculoskeletal Function Assessment (MFA)*The MFA questionnaire is a 100‐item self-reported health status instrument designed by Martin et al. (71) for use in a broad range of patients with musculoskeletal disorders of the extremities. Scores can range from 0 to 100, with a higher score indicating poorer function. It has been found both valid and reliable in five musculoskeletal disorders of the upper and lower extremities (fractures, soft-tissue injuries, repetitive motion disorders, osteoarthritis, and rheumatoid arthritis).

*Short Musculoskeletal Function Assessment (SMFA)*The SMFA questionnaire is a shorter version of the MFA and was developed by Swiontkowski et al. (REF) and consists of 46 items. It was designed to assess the functional status of patients with various musculoskeletal disorders and injuries. The SMFA items are divided over two indices: “function index” and “bother index” (72). In the Dutch version the items are additionally structured into four subscales (lower extremity dysfunction, upper extremity dysfunction, problems with daily activities and mental and emotional problems). The scoring system is similar to that of the MFA. The SMFA has been proven to be a valid and reliable questionnaire and the Dutch version has also been shown to be valid and reliable for patients sustaining injuries (73). *Short-Form-36 (SF-36)*
The SF-36 (74) and is a widely used valid and reliable instrument to measure health-related quality of life. It is subdivided into eight scales: physical functioning (PF), role physical (RP), bodily pain (BP), general health (GH), vitality (VT), social functioning (SF), role emotional (RE), and mental health (MH). Score on each subscale is transformed into a 0-100 point scale, with a higher score indicating less disability. Additionally, scores from the subscales can be aggregated in two distinct, higher-order summary scores:: the Physical Component Summary (PCS) representing the physical dimension and the Mental Component Summary (MCS) representing the mental dimension.

*Short-Form-12 (SF-12)*The SF-12 (75) is derived from the SF-36 and is a multipurpose generic measure of health status. This 12-item questionnaire was designed to reduce respondent burden while achieving minimum standards of precision monitoring health in general and in specific populations. Similar to the SF-36, it measures eight health aspects and the same two summary scales (PCS and MCS) can be calculated. It has been found valid and reliable for multiple health conditions.

*EuroQuol-5D (EQ-5D)*
The EQ-5D is a 5-item questionnaire that measures health-related quality of life based on five dimensions of health: mobility, self-care, daily activities, pain/discomfort and anxiety/depression (76). Patients can use the dimensions to delineate to what extent they experience problems, scoring from 1 (no problems) up to 5 (extreme problems). The combination of scores on the 5 dimensions are converted to a utility value that ranges from ≤0 to 1, in which a higher score indicates a better quality of life. The EQ-5D is widely used and found to be both valid and reliable in a wide range of conditions and populations.
