# Supplementary material for: Patient-reported physical functioning and quality of life after pelvic ring injury: A systematic review of the literature
Source: PLoS One. 2020 Jul 17;15(7):e0233226. doi: 10.1371/journal.pone.0233226 (PMC7367481; doi:10.1371/journal.pone.0233226)
Supplement: Attachment — Submitted filename: Response to reviewers.docx [file pone.0233226.s003.docx]

**Response to reviewers**

Dear members of the editorial board,

Thank you for your important remarks on our manuscript. We will address each of your points in the following section:

**1. In terms of your results, what is the single most useful general and specific PROM you recommend to use in pelvic ring injuries**
Until there is a validated pelvic-specific PROM, we can only recommend to use a validated general PROM to evaluate the outcomes after these injuries (lines 391-394). We favour the SMFA for evaluation of physical functioning because it is compact, valid and reliable. For quality of life we recommend the EQ-5D or the SF-36 which are both valid and reliable, widely used, and often with available normative data to compare with.

**2. Although your manuscript involved a lot of papers in the literature, however it did not differentiate between patient groups. For example: are the papers included in the systematic review included polytrauma patients or not? Also are the papers included differentiate between geriatric pelvic fractures and other age groups? As these 2 variables may affect the patient outcomes**
Thank you for this remark. The included papers could also include patients with polytrauma, since we included all studies with patients over 18 years old with a pelvic ring injury. Most pelvic injuries are seen together with other injuries (with the exception of geriatric fractures), as the cause is often a high-energy trauma. These indeed affect the outcomes, but studies sadly did not differentiate between outcomes of polytrauma patients or patients with a pelvic ring injury as a single injury. This is a very important remark for future research and we added this in the manuscript as part of the discussion (lines 286-288) and of the recommendation (lines 386-387).
We excluded patients with geriatric fractures or fragility fractures of the pelvis, because the results in terms of PROMs do not seem comparable with the PROMs of patients with normal or good bone quality. This exclusion was not shown in the manuscript, therefore we added this in the in- and exclusion criteria (line 108).

We hope to have answered all of your questions and managed to correctly address all of your remarks in the revised manuscript.

Hester Banierink,

On behalf of all authors
