# Supplementary material for: Patient-reported physical functioning and quality of life after pelvic ring injury: A systematic review of the literature
Source: PLoS One. 2020 Jul 17;15(7):e0233226. doi: 10.1371/journal.pone.0233226 (PMC7367481; doi:10.1371/journal.pone.0233226)
Supplement: Attachment — Submitted filename: Response to reviewers.docx [file pone.0233226.s004.docx]

**Response to reviewers**

Dear members of the editorial board,

Thank you for your important remarks on our manuscript. We will address each of your points in the following section:

**Methods section**

1. **Page 11, line 95: please write inclusion instead of “In.”**

Done

**Results section**

1. **All case either treated with operative or non-operative treatment ways were included, there are no data presented to compare between those types of patients regarding PROMs**
   This is right. In this study we wanted to provide an overview of all available outcomes and not compare types of treatment. Of course this would be interesting, but with all the different operative techniques that were used, as well as outcome measures, the results of this comparison would not be valid.
2. **Page 13, line 157: in the sentence (conducted January 2008 to April 15th 2019) write “from” after conducted.**

Done

3. **line169: write the reference after “ AO classification system”**

Done

4. **It’s better to insert the tables of results in the main manuscript better than additional files, as they are an integral part of the manuscript**

We agreed and added the tables in the main manuscript

**Limitations section**

1. **Please include the non-calculation of the sample size in all studies.**

Done

2. **Also, gathering of data with multiple tools of assessment is a major limitation.**

This is also added in the limitations section, together with the fact that it includes all types of pelvic ring injuries and treatment methods.

We hope to have answered all of your questions and managed to correctly address all of your remarks in the revised manuscript.

Hester Banierink,

On behalf of all authors
